# Emotional modulation of the attentional blink and the relation to interpersonal reactivity

**DOI:** 10.3389/fnhum.2013.00641

**Published:** 2013-10-11

**Authors:** Philipp Kanske, Sandra Schönfelder, Michèle Wessa

**Affiliations:** ^1^Department of Social Neuroscience, Max Planck Institute for Human Cognitive and Brain SciencesLeipzig, Germany; ^2^Department of Clinical Psychology and Neuropsychology, Johannes Gutenberg UniversityMainz, Germany

**Keywords:** P3 event-related potential, electroencephalography, event-related potentials, empathy, attentional blink, emotions

## Abstract

The extent of the attentional blink effect on detection rates in rapid serial visual presentations is modulated by the emotionality of the stimuli. Emotionally salient stimuli are detected more often, even if presented in the attentional blink period, and elicit an enlarged P3 response, which has been interpreted as enhanced consolidation. This effect correlates with individual differences in trait affectivity such as anxiety or dysphoria. Here, we ask if it is also related to the capacity to detect emotions in others, i.e., to interpersonal social traits. We therefore presented emotional and neutral images depicting social scenes as targets in an attentional blink design and measured detection rates and event-related potentials. In addition, we recorded self-reports of empathy as measured by the Interpersonal Reactivity Index. The results show enhanced performance for emotional stimuli and increased P3 amplitudes, which correlated with individual differences in empathy. The data suggest that self-reported empathy goes along with enhanced processing of emotion in social stimuli, even under stimulus conditions that are suboptimal for conscious target detection.

## INTRODUCTION

The attentional blink phenomenon occurs when two stimuli are presented briefly one after the other, with the first stimulus impairing processing of the second stimulus because attentional resources cannot be sufficiently allocated (Raymond et al., 1992). The attentional blink effect is most widely observed in rapid serial visual presentation paradigms when the two target stimuli that need to be identified are embedded in a stream of distractor stimuli (Martens and Wyble, 2010). While identification of the first target (T1) is typically not affected, performance on the second target (T2) is impaired, if it is presented about 200 to 500 ms after the first target, but not in earlier or later time windows. The critical question regarding the stages at which processing of the second target is interrupted has been thoroughly addressed with the help of event-related potentials (ERPs) of the electroencephalogram. These data showed that the N1, P1, and N400 components of the ERP in response to T2 are not altered, which suggests that early sensory processing and semantic analysis of T2 are intact (Vogel et al., 1998). The P3 component however is typically found to be reduced for non-identified T2, which has been interpreted as impaired consolidation of the stimulus in working memory (Rolke et al., 2001; Vogel and Luck, 2002; Kranczioch et al., 2003). The suggestion that the P3 indexes working memory processes is already relatively old and based on data showing its sensitivity to the probability of task-defined stimulus categories (Donchin, 1981; Donchin and Coles, 1988). Nevertheless it is still consistent with the accumulated evidence, for example, through manipulations of memory load or subsequent recognition (for a review on P3 function, see Polich, 2007).

The size of the attentional blink effect is influenced by a number of different factors including personal relevance (Shapiro et al., 1997) and emotionality of the target stimuli (Anderson and Phelps, 2001). If the second target is emotionally negative or positive, the attentional blink is reduced, such that more of these stimuli are detected than neutral targets. In a series of experiments, Keil and Ihssen (2004) showed that this effect is related to emotional arousal rather than valence. In line with these behavioral effects, the amplitude of the P3 has been found to be enlarged in response to correctly identified emotional T2 stimuli (Trippe et al., 2007). These results have been interpreted as preferential selection of affective information, facilitating working memory consolidation (Keil and Ihssen, 2004).

Inter-individual variations in the extent to which emotions influence the attentional blink effect have also been repeatedly observed, particularly for individual differences in trait anxiety (Fox et al., 2005; Van Dam et al., 2012) or dysphoria (Koster et al., 2009). Further, the attentional blink effect is altered in mental disorders with clinically relevant changes in emotion processing, such as specific phobia (Trippe et al., 2007) or post-traumatic stress disorder (Amir et al., 2009). These correlations speak to the influence of a participant’s own affect on the processing of emotional stimuli embedded in a rapidly presented visual stream. However, it remains an open question whether the emotional modulation of the attentional blink also relates to individual differences in the capacity to react to emotions in others (i.e., in interpersonal social traits). The term empathy has often been used in a broad sense, encompassing multiple facets of interpersonal reactivity, as in the frequently used Interpersonal Reactivity Index (IRI; Davis, 1983b), which asks for trait capabilities in emotional and more cognitive reactions towards others. Enhanced processing of emotional stimuli may be a basis for empathic reactions as the identification of the emotional content of a social scene necessarily precedes a reaction on the side of the observer. Interestingly, empathy has also been related to social reward sensitivity and social attention, which might mediate this relation. In a study of 8–12-year-old children, parent-reported empathic skills were related to behavioral benefits due to social reward, but not to monetary reward (Kohls et al., 2009). Data from individuals diagnosed with high functioning autism who showed deficits in empathic skills also indicates reduced sensitivity to social, but not monetary reward (Demurie et al., 2011; Delmonte et al., 2012). Probably related to this, autism is also characterized by drastically reduced attention to social stimuli and a preference to attend to non-social objects (for a review, see Dawson et al., 2012). Better differentiation of social stimuli could be a mechanism that enables them to be perceived as rewarding and to be attended more, which in turn allows for empathic reactions. There is already some indication that the reactivity to emotional stimuli correlates with empathy as measured by the IRI (Davis, 1983b). Silani et al. (2008) reported that in a group of individuals with high functioning autism an increase in activity of the amygdala in response to emotional images was related to empathy scores. Similarly, empathy scores correlated with amygdala responses to emotional faces in a developmental study of 10-year-old children (Pfeifer et al., 2008). The present study aims at extending these results to the attentional blink paradigm and tests healthy adults with a wider age range. The attentional blink task offers the advantage of testing emotion detection under stimulus conditions that are suboptimal for conscious target detection. Thereby it allows us to address the important question whether, under difficult stimulation conditions and high working memory load, individuals scoring high in empathic traits are more sensitive to emotional expressions and process these more deeply than those scoring low in empathy. We hypothesized that this difference is reflected in amplitude modulations of the P3, indicating enhanced consolidation of emotional T2 stimuli in high empathic individuals.

To this end, we used an attentional blink paradigm with emotional and neutral images as T2. They always followed neutral T1 stimuli and were embedded in streams of neutral distractors. The depicted scenes to be analyzed were always “social” in that humans were displayed. During the task, an electroencephalogram was recorded. In addition, we acquired the IRI (Davis, 1983b) from each participant and correlated the respective sum and subscale scores to the emotional modulation of the attentional blink effect. We expected (1) to find the behavioral attentional blink effect in lower T2 than T1 recognition rates, (2) a modulation of this effect by emotion such that emotional T2 are recognized more often than neutral T2, (3) a reflection of this effect in the ERP with larger P3 amplitudes to emotional than neutral T2 and (4) a positive correlation of this effect to inter-individual differences in empathy scores acquired with the IRI (Davis, 1983b). As only few studies have investigated the emotional modulation of the attentional blink with pictures (instead of written words, for example, Trippe et al., 2007) and mainly used objects rather than social scenes for the neutral condition, the results of the present study with social scenes in neutral and emotional conditions will allow broader generalizability, which is particularly relevant for testing its relation to interpersonal social traits.

## METHODS

### PARTICIPANTS

Twenty-seven healthy individuals (16 females) between 19 and 56 years (mean age = 31.07 years, SD = 11.13) took part in this study. All participants were native German speakers, right-handed according to the Edinburgh handedness inventory (Oldfield, 1971), and had normal or corrected-to-normal vision. Prior to their enrollment in this study, each participant was screened by telephone by an experienced clinical psychologist for exclusion criteria that included current or lifetime mental disorders, visual or hearing impairments, a lifetime history of head injury with loss of consciousness, brain damage or surgery, the presence of a cardiovascular disease, neurological illness, and regular use of medication (except for oral contraceptives). The presence of mental disorders, including alcohol or drug abuse, was evaluated by screening items that relied on the key diagnostic questions from the Structured Clinical Interview for DSM-IV-TR Axis I Disorders (SCID-I; German version: Wittchen et al., 1997). The study protocol was approved by the Ethics Committee of the Medical Faculty Mannheim, Heidelberg University, and written informed consent was obtained from each subject prior to the experimental session.

### INTERPERSONAL REACTIVITY INDEX

Subjects filled out the German translation of the IRI (original English version: Davis, 1980). This 28-item questionnaire assesses empathy in the form of statements that have to be agreed or disagreed on a Likert-type scale ranging from 1 (“does not describe me well”) to 5 (“describes me very well”). The IRI can be subdivided into four subscales, each comprising seven items: “Empathic concern” and “Personal distress” refer to the two affective empathy dimensions that measure respondents’ other-oriented feelings of compassion, warmth and concern for unfortunate others, and respondents’ self-related discomfort and anxiety arising from observing other people’s suffering, respectively. The subscales “Perspective taking” and “Fantasy” determine more about the cognitive empathy domain (Shamay-Tsoory et al., 2009) and inquire about the ability to mentally adopt the perspective of others as well as the tendency to identify with characters in fictional situations (e.g., movies and novels), respectively. Typically, an overall IRI score is calculated as index of the general capacity to empathize with others. The IRI subscales of the original American version (Davis, 1980) possess good psychometric properties with Cronbach’s α coefficients of internal consistency ranging from 0.71 to 0.77 and test–retest reliability coefficients ranging from 0.61 to 0.81 (Davis, 1980). The basic psychometric quality of our German translation of the IRI was comparable in the present sample with satisfying Cronbach’s α coefficients (i.e., 0.79 for “Fantasy,” 0.82 for “Perspective taking,” 0.78 for “Personal distress,” and 0.68 for “Empathic concern”).

### STIMULUS MATERIAL

The computerized attentional blink task comprised picture stimuli selected from the International Affective Picture System (IAPS; Lang et al., 2005), a standardized in-house set of emotionally evocative pictures (Emotional Picture Set (EmoPicS); Wessa et al., 2010) and public internet photo libraries. The T2 stimuli were separated into three affective categories that all portrayed humans (see **Table 1** for mean valence and arousal ratings for the stimulus selection). Ten pictures depicted negative scenes of human violence, mutilation, loss, and illness, 10 neutral pictures showed human faces or people doing ordinary activities, and 10 pictures displayed positive scenes including happy families, erotic couples, and exciting sports. Based upon the normative data provided for the IAPS and EmoPicS databases, the emotional categories differed statistically with respect to valence [*F*(2,87) = 1683.06; *p* < 0.001] and arousal [*F*(2,87) = 214.75; *p* < 0.001]. To minimize sex differences (cf. Bradley et al., 2001), only pictures with relatively small gender differences in normative ratings (within 1.5 points on the 9-point scales for both affective valence and arousal) were included. To control for physical picture parameters, luminance, contrast, and color composition (red, blue, and green layer) values were extracted for each image using the histogram function of Adobe PhotoShop^©^ software (version 9.0; Adobe Systems Inc., San Jose, CA, USA). A subsequent multivariate analyses of variance (ANOVA) performed on these measures did not reveal any significant differences between the categories [luminance: *F*(2,87) = 1.16; *p* = 0.318, contrast: *F*(2,87) = 1.13; *p* = 0.327, red layer: *F*(2,87) = 0.79; *p* = 0.457, green layer: *F*(2,87) = 1.15; p = 0.320, blue layer: *F*(2,87) = 2.77; *p* = 0.068]. The T2 stimuli were repeated such that there were 5 presentations of each neutral, positive, and negative image of a human as T2, leading to a total of 150 trials. Additionally, 150 trials with T2 stimuli depicting plants and animals were presented, each category appearing in 75 trials. These two semantic categories were added in order to reduce the chance level identification rate of T2 stimuli but were not part of the statistical data analysis. Overall, 40 neutral pictures of plants and 40 neutral pictures of animals served as stimuli for T1 and T2. Ten additional neutral pictures of humans were incorporated as T1. The attentional blink experiment comprised 300 trials with pictures of animals, plants, and humans occurring equally often as targets (T1 and T2). More specifically, each category was presented 200 times as target, such that human images appeared in 50 trials as T1 stimulus and in 150 trials as T2 (i.e., with 50 trials per emotion category), whereas plants and animals were shown in 125 of all trials as T1, and in 75 trials as T2, respectively. Finally, distractors encompassed 45 additional neutral pictures that depicted a wide range of plants, animals, and humans.

**Table 1:** Mean valence and arousal ratings and standard deviations (in parentheses) for T2 images.

Normative IAPS and EmoPicS ratings		Sample ratings	
Valence	Arousal	Valence	Arousal
Negative	2.02 (0.48)	6.71 (0.82)	2.23 (0.63)	5.81 (1.31)
Neutral	5.01 (0.36)	3.16 (0.35)	5.18 (0.28)	1.98 (0.88)
Positive	7.15 (0.32)	6.30 (0.62)	6.98 (0.86)	4.76 (1.86)

*Normative IAPS and EmoPicS ratings and the ratings of the present sample are displayed.*

### EXPERIMENTAL PARADIGM AND PROCEDURE

**FIGURE 1:**
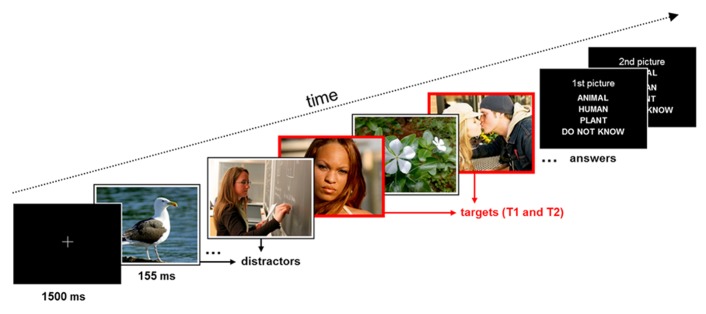
**Schematic of the trial structure and time course of stimulus events in the experiment.** Participants were requested to identify the content of the two red-framed target pictures (T1 and T2; time interval between targets 310 ms) within a rapid series of white-framed distractor pictures.

During the attentional blink paradigm, participants were asked to identify two target images (T1 and T2), which were present in a stream of distractor images. The second target (T2) was always presented shortly (310 ms) after the first (T1), so that it fell in the attentional blink period (Kranczioch et al., 2003). The sequence of events in a trial (see **Figure 1**) was the following: after the presentation of a fixation cross for 1500 ms, a stream of 2–10 distracter images indicated by a white frame was presented. Each of these images was presented for 155 ms. A first target image (T1), indicated by a red frame, was shown next, followed by a single distractor image, the second target image (T2), and another 10 distractor images. At the end of each trial participants saw two successive response screens asking whether T1 and T2 showed (1) an animal, (2) a human, (3) a plant, (4) or if they did not know the answer. They responded via button press with the right hand. The response screens were presented until the participant pressed a button. The total number of trials was 300. These were split up into five blocks with short breaks. The experiment lasted about 45 min.

### EEG DATA ACQUISITION AND ANALYSIS

Electroencephalography activity was recorded from 60 scalp sites (Fpz, Fz, Fcz, Cz, Cpz, Pz, POz, Oz, Fp1/2, AF3/4, AF7/8, F1/2, F3/4, F5/6, F7/8, FT7/8, Fc1/2, FC3/4, FC5/6, T7/8, C1/2, C3/4, C5/6, CP1/2, CP3/4, CP5/6, TP7/8, P1/2, P3/4, P5/6, P7/8, PO3/4, PO7/8, and O1/2), placed according to the extended International 10–20 system (Jasper, 1958; American Electroencephalographic Society, 1991), with Ag/AgCl-sintered electrodes and a ground positioned on AFz. Horizontal and vertical electroocular activity (EOG) was measured using a bipolar configuration lateral at the outer canthi of both eyes and from above and below the right eye. The right mastoid served as online reference. Electrode impedances were kept below 10 kΩ. Raw EEG signals were continuously registered with a sampling rate of 500 Hz (DC; 1000 Hz high-frequency cut-off) through two BrainAmp amplifiers (Brain Products GmbH, Munich, Germany).

Offline, data analysis was performed with Brain Vision Analyzer 2 software (Version 1.05; Brain Products GmbH, Munich, Germany). EEG data were initially re-referenced against the algebraic mean of the left and right mastoids and digitally filtered with a 0.1–25 Hz (48 dB/octave) bandpass filter. Eyeblink and horizontal ocular artifacts were subsequently corrected by an independent component analysis algorithm. Continuous EEG signals were segmented separately for positive, negative, and neutral T2 pictures into 2000 ms epochs (for the time period of 500 ms pre-stimulus to 1500 ms post-stimulus onset). All trials were semiautomatically screened for technical, muscle-related, or movement-related artifacts with amplitude deviations of ±80 μV and corrected relative to the 500 ms pre-stimulus baseline interval. In addition, trials were visually inspected and excluded if further artifacts were visible (e.g., extreme alpha activity). ERPs were obtained by averaging trials separately for each subject, electrode site and T2 category (positive, neutral, negative) and for all trials where T2 was (a) correctly identified or (b) incorrectly identified or the answer choice “do not know,” indicating that the target stimuli was not identified, was selected. ERPs to T2 presentation were only included in the analysis when the preceding T1 image had been correctly identified. Magnitudes of the P3 component were extracted from these averaged waveforms as mean activity in the pre-determined time interval of 300–800 ms after T2 onset from nine electrode sites (F3, Fz, F4, C3, Cz, C4, P3, Pz, and P4).

### STATISTICAL ANALYSES OF BEHAVIORAL DATA

Accuracy was analyzed with PASW (version 15.0, SPSS Inc., Chicago). A repeated-measures ANOVA with the factor emotion (negative, neutral, positive) was computed to elucidate the effects of emotion on picture recognition in the attentional blink. Only those trials in which T1 was correctly identified were included (see e.g., Trippe et al., 2007). Repeated pair-wise comparisons with Bonferroni correction were computed to test the differences between negative, neutral, and positive trials. All effects with a *p* < 0.05 were treated as statistically significant. Greenhouse-Geisser corrections were applied to significant *F* ratios that did not meet Mauchly’s sphericity assumption. Only interactions that yielded significant follow-up analyses are reported.

Further, we computed Pearson product-moment correlations between the IRI sum score and the attentional blink performance data as well as the P3 effect. Significant correlations with the composite IRI score were followed up by *post-hoc* correlations for the four IRI subscales. In order to quantify the P3 effect, we calculated difference scores by subtracting the ERP activity elicited by neutral images from the activity elicited by each emotion category (i.e., positive-neutral and negative-neutral) in the 300–800 ms time window and averaged across the analyzed nine electrode locations. The correlations were one-tailed because we had directional hypotheses (higher IRI scores are positively associated with the P3 increase for emotional T2). Correlations of the IRI subscales were treated as statistically significant when surviving a Bonferroni corrected *p*-value of *p* < 0.0125 (*p* < 0.05/4 IRI subscales).

## RESULTS

### BEHAVIORAL RESULTS

**FIGURE 2:**
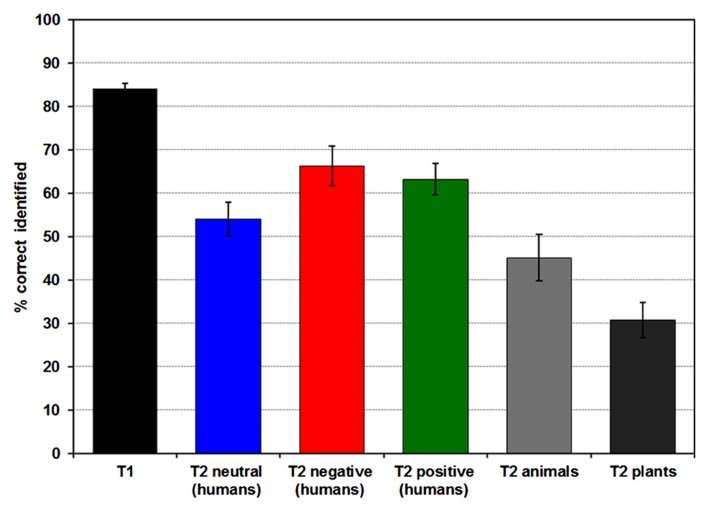
**Diagram of mean percentage (and standard error) of correctly identified T1 and T2 pictures.** T2s were only included when the preceding T1 was detected correctly.

A total of 84.15% (SD = 6.26) of all T1 images were correctly identified, correct T2 identification rate (after correct T1 identification) was 51.88% (SD = 19.39) collapsed over all image categories (see **Figure 2**). Emotion significantly influenced T2 recognition rates [*F*(2,50) = 14.251, *p* < 0.001, partial ŋ^2^ = 0.363; positive_mean (SD)_ = 64.24% (18.64), neutral_mean (SD)_ = 55.13% (20.46), negative_mean (SD)_ = 66.28% (23.44)], with higher recognition accuracy in negative and positive compared to neutral T2 images (both *p’s *= 0.001). T2 recognition rates for positive and negative pictures did not differ (*p* > 0.15).

### ERP RESULTS

**FIGURE 3:**
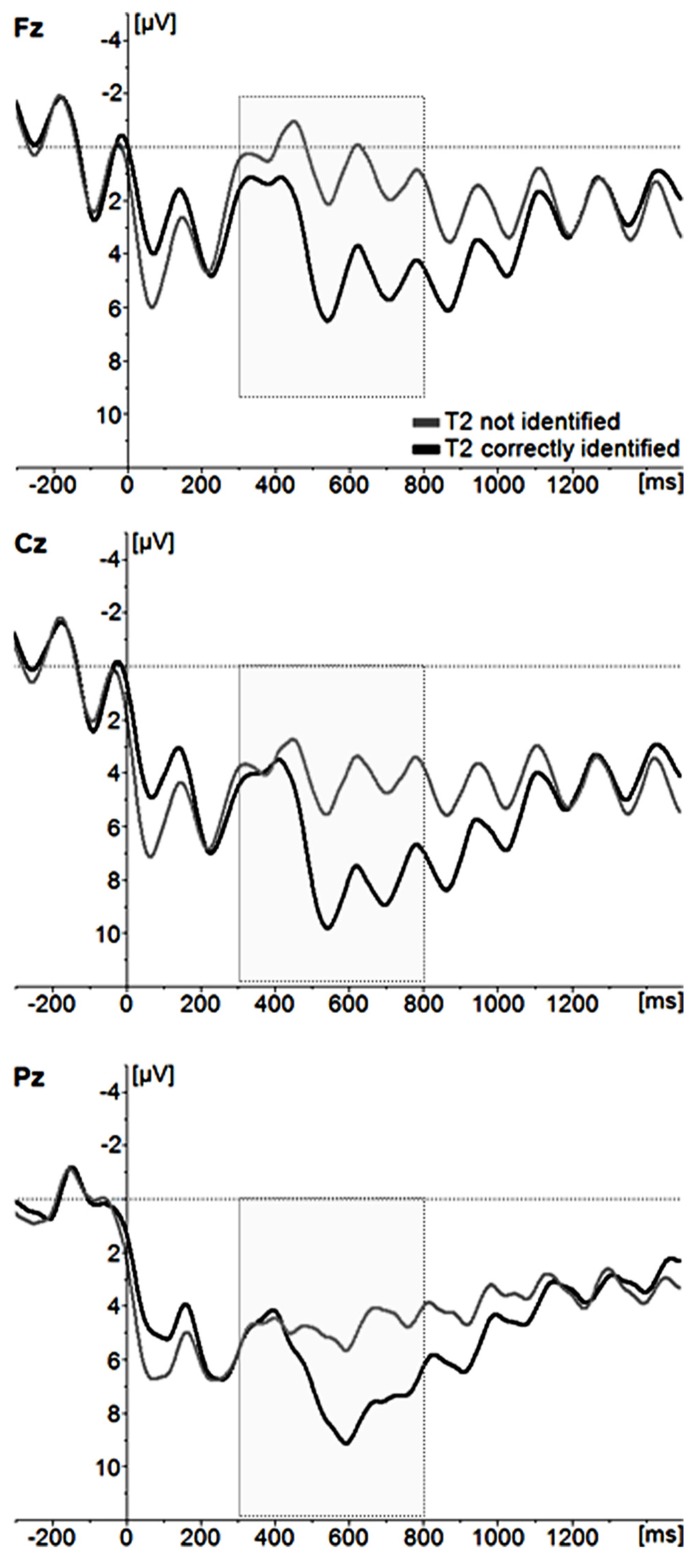
**Grand average ERP waveforms for incorrectly identified and correctly identified T2 images.** For illustrative purposes, only the midline electrode sites Fz, Cz, and Pz are displayed.

Stimulus-locked ERP waveforms for correctly and incorrectly identified T2s following correctly identified T1 stimuli are displayed in **Figure 3**. Here, a repeated measures ANOVA with T2 identification accuracy (correct, incorrect) and two electrode site factors [lateralization (left, central, right); anteriority (anterior, central, posterior)] were calculated. All included participants had ≤10 trials for each condition. In accordance with previous research (e.g., Katayama and Polich, 1999), the scalp topography of the P3 showed a maximum voltage change over centro-parietal electrode sites [main effect of electrode site; anteriority *F*(2,52) = 50,652, *p* < 0.001, partial ŋ^2^ = 0.661; anterior_mean_ = 0.556 μV, central_mean_ = 3.750 μV, posterior_mean_ = 4.521 μV; laterality *F*(2,52) = 7,786, *p* < 0.001, partial ŋ^2^ = 0.230; left_mean_ = 4.418 μV, central_mean_ = 4.434 μV, posterior_mean_ = 3.680 μV]. The analysis also showed that correctly identified images elicited a larger P3 amplitude than incorrectly identified images [*F*(1,26) = 15.560, *p* < .001, partial ŋ^2^ = 0.374].

**FIGURE 4:**
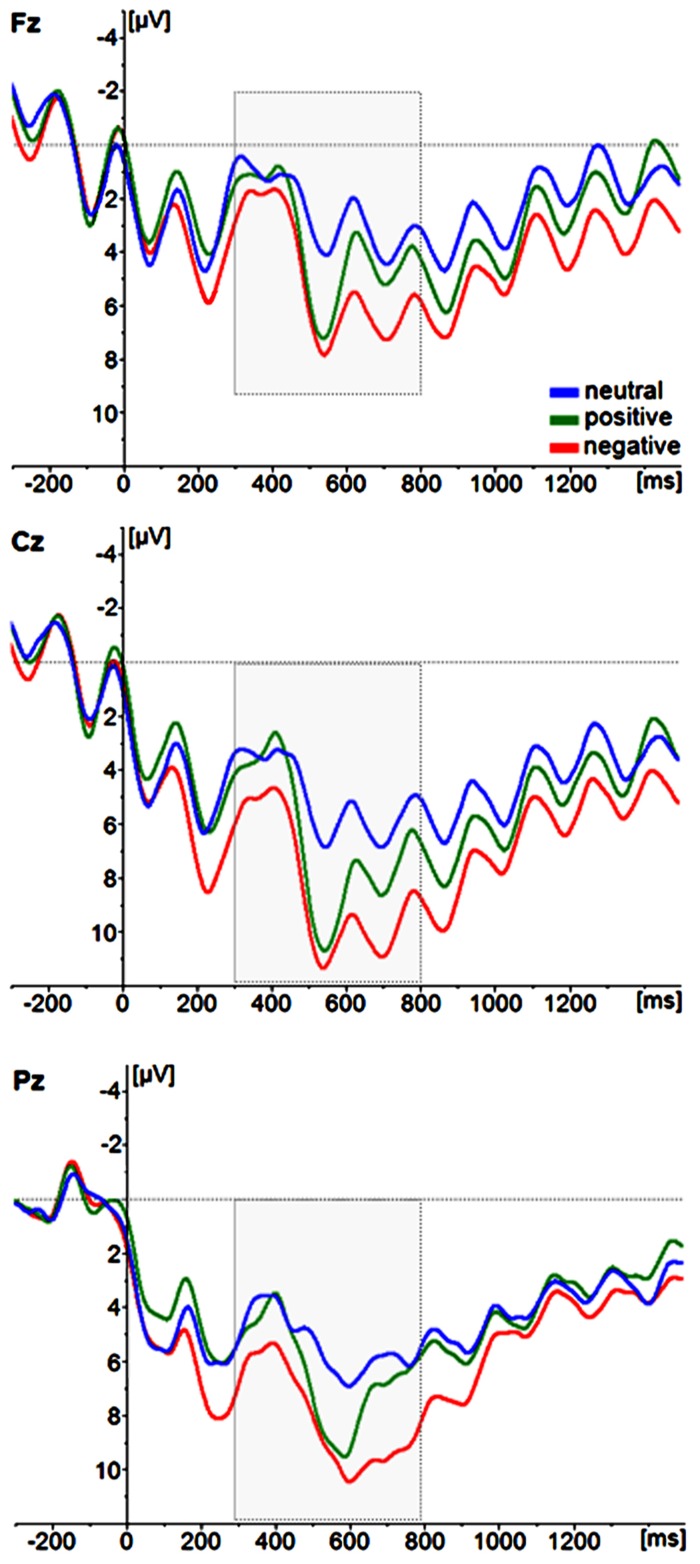
**Grand average ERP waveforms of correctly identified T2 image categories (neutral, positive, negative).** For illustrative purposes, only the midline electrode sites Fz, Cz, and Pz are displayed.

ERPs for trials with correctly identified positive, neutral, and negative T2s are depicted in **Figure 4**. To evaluate the effects of T2 emotion category on ERP magnitudes, a repeated-measures ANOVA with the within-subject factors emotion category (positive, negative, neutral) and the same electrode site factors as above was performed. Again, the topography of the P3 showed a maximum over centro-parietal electrode sites [main effect of electrode site; anteriority *F*(2,52) = 18,138, *p* < 0.001, partial ŋ^2^ = 0.411; anterior_mean_ = 5.3626 μV, central_mean_ = 8.534 μV, posterior_mean_ = 7.755 μV; laterality *F*(2,52) = 14,387, *p* < 0.001, partial ŋ^2^ = 0.356; left_mean_ = 7.200 μV, central_mean_ = 7.947 μV, posterior_mean_ = 6.504 μV]. Critically, the magnitude of the P3 amplitude differed as a function of emotion category [*F*(2,52) = 16.783, *p* < 0.001, partial ŋ^2^ = 0.392; Cz: positive_mean (SD)_ = 10.55 μV (6.05), neutral_mean (SD)_ = 6.56 μV (3.84), negative_mean (SD)_ = 11.07 μV (5.53)], with *post-hoc* pairwise comparisons indicating that the mean P3 amplitude to highly arousing positive and negative pictures differed from the P3 amplitudes to neutral pictures (negative vs. neutral: *p* < 0.001; positive vs. neutral: *p* < 0.001), but not from each other (negative vs. positive: *p* = 1.00). There were no interactions with the electrode site factors (all *p* > 0.10). There were also no effects of emotion category in the incorrectly identified T2 images (*p* > 0.05).

### CORRELATIONS OF EMPATHY QUESTIONNAIRE DATA WITH ATTENTIONAL BLINK PERFORMANCE AND ERP ACTIVITY

**FIGURE 5:**
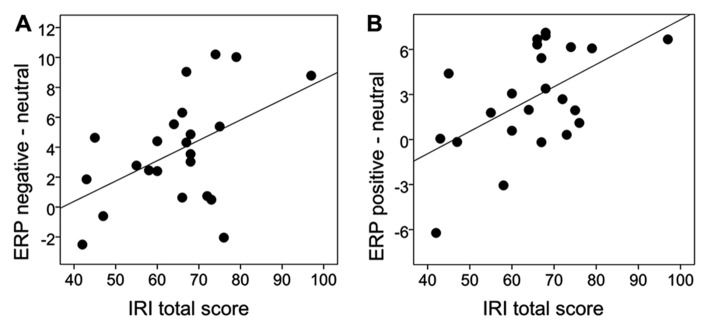
**Scatterplot of the correlation between the P3 effect and IRI total score.** The P3 effect was quantified as the difference in the ERP waveform activity between emotional and neutral T2 pictures (i.e., negative-neutral, positive-neutral) in the time window of 300–800 ms.

**Table 2** summarizes the empathy questionnaire scoring results of the present sample. For the behavioral attentional blink data we examined correlations between empathy and the emotional attentional blink effect by calculating difference scores of accuracy between the neutral and emotional picture categories (i.e., negative-neutral, positive-neutral). Neither positive nor negative images correlated significantly with the IRI total score (negative-neutral: *r* = -0.07, *p* = 0.373, positive-neutral: *r* = -0.23, *p* = 0.147). Hence, no further correlations were calculated. To correlate empathy to the P3 effects, we averaged across the analyzed electrodes and formed difference scores of negative-neutral and positive-neutral conditions. For the negative-neutral condition there was a significant correlation with the IRI total score (*r* = 0.49, *p* = .009). Similarly, there was a correlation for the positive-neutral condition (*r* = 0.55, *p* = .003), see **Figure 5**. Due to significant correlations with the IRI total score, we further examined the relation of P3 effects to the IRI subscales we found significant correlations for “fantasy” scores (with negative-neutral: *r* = 0.52, *p* = 0.005 and with positive-neutral: *r* = 0.61, *p* = 0.001) and for “perspective taking” scores (with negative-neutral: *r* = 0.54, *p* = 0.004 and with positive-neutral: *r* = 0.77, *p* = 0.000009). Analysis of “empathic concern” and “personal distress” scores did not reveal a significant correlation with P3 effect size (all *p*’s > 0.25).

**Table 2:** Scores on the empathy questionnaire.

Scale	M	SD	min	max
Perspective taking	18.22	4.88	6	28
Empathic concern	18.83	4.07	11	25
Fantasy	15.56	5.57	2	24
Personal distress	12.17	5.17	4	25

## DISCUSSION

The present study showed a correlation of the P3 increase to emotional stimuli presented in the attentional blink with inter-individual differences in empathy as measured through self-reports. This suggests that individuals scoring high in interpersonal social traits show enhanced processing of emotional stimuli under stimulus conditions that are suboptimal for conscious target detection and high working memory load. The study also corroborates our knowledge about the attentional blink phenomenon by showing larger P3 amplitudes to correctly identified T2 stimuli and through demonstrating that emotional picture stimuli are detected more often, even if they are presented in the attentional blink period. Because of the very limited amount of attentional blink studies with pictorial stimuli and socio-emotional scenes in particular, the study also extends the phenomenon to more ecologically valid stimuli.

The influence of emotion on the attentional blink was apparent in the increased detection rates for emotional pictures and in the enlarged P3 amplitude to emotional T2 images (cf. Anderson and Phelps, 2001; Keil and Ihssen, 2004; Trippe et al., 2007). However, a correlation of the emotion effect with empathy scores (Davis, 1983b) was only observed in the P3 amplitude, not for the behavioral performance. Thus, individuals with high scores on empathy did not differ from participants scoring low on empathy in the percentage of correctly identified emotional targets relative to neural targets. P3 responses to those emotional images that were correctly identified, however, were enlarged. This pattern could suggest a dissociation of behavioral and ERP responses in the attentional blink, which has already been reported by Trippe et al. (2007) who noted that they seem to be influenced through at least partially different neural processes. While the P3 is clearly linked to working memory consolidation, error rates represent a composite of multiple processing stages also including sensory processing and response preparation. This may yield the P3 potential a more sensitive measure and the present data suggest that particularly working memory consolidation is modulated by trait empathy (Vogel et al., 1998; Vogel and Luck, 2002).

The fact that the correlation pattern was observed for both positive and negative emotional images corroborates the findings for two different categories of stimuli. A similar influence of positive and negative emotional stimuli on the attentional blink (Keil and Ihssen, 2004; Ogawa and Suzuki, 2004; Trippe et al., 2007) and other attentional processes (Brosch et al., 2008; Kanske and Kotz, 2011a,b; Kanske, 2012) is in line with previous work and shows that putative reward and threat signals can possess similar salience. The present correlations indicate that the sensitivity of people scoring high in empathy applies to both positive and negative emotion in facial expressions and scenes. While it may be argued that such a relation is principally self-evident, the association of ERP with self-report data on how individuals habitually perceive themselves in reaction to others is noteworthy. Furthermore, the present data can specifically link this association to a particular processing stage, namely consolidation in working memory associated with the P3 component (Vogel and Luck, 2002; Martens and Wyble, 2010).

An interesting aspect of the present data is the specific correlation pattern between the IRI subscales and the emotional modulation of the attentional blink. In addition to the total score, the subscales fantasy and perspective taking were also individually correlated with the effect, while personal distress and empathic concern showed no significant correlation. Even though we had no a priori hypotheses regarding the subscale correlation, one might have expected to find a relation between all subscales, in particular personal distress and empathic concern, as they have been interpreted as representing the more affective aspect of the empathy construct (Davis, 1983a,b). However, it may be that the correlational pattern with the more cognitive, but not affective scales reflects the fact that the P3 indexes a rather late stage of stimulus processing, i.e., working memory consolidation. Possibly, earlier stages related to sensory processing and first classification of stimuli would show a different pattern, namely a correlation with the affective subscales of the IRI (Yamada and Decety, 2009; Decety et al., 2010). The present paradigm was not optimized to look at ERP components indexing these stages, as would for example an emotional dot probe paradigm (Pourtois et al., 2005). We did not observe such effects, but future research could address this point with more specialized designs. Nevertheless, it is also important to note that previous studies also reported correlation of the more cognitive subscale perspective taking to emotional reactivity as measured in amygdala activity (for example, Pfeifer et al., 2008; Silani et al., 2008). A more promising path to differentiating cognitive and affective social understanding may lie in assessing them experimentally, rather than with self-reports (Dziobek et al., 2006, 2008; Apperly et al., 2011). Performance on respective tasks could yield objective performance levels that are not subject to the biases inherent in self-report data.

There are some limitations to the present study. As already outlined, a more comprehensive insight into the relations of the presently discussed concepts could be gained by assessing emotional reactivity in more than one paradigm to allow better description of multiple stimulus processing stages (for example, Kanske et al., 2011). Additionally, applying multiple measures for empathy, in addition to self-report data, would potentially better separate cognitive and affective aspects (Shamay-Tsoory et al., 2009; Harari et al., 2010). A critical point regarding the presently used paradigm is that emotional stimuli are presented less frequently than neutral stimuli, in particular when considering the amount of distractor images. Thus, the P3 modulation and the better detection rates may just reflect a saliency through infrequency effect. While this is an inherent problem of all studies looking at an emotional modulation of the attentional blink, several studies reported differential effects for different discrete emotions, such as fear, anger, or happiness (Fox et al., 2005; Maratos et al., 2008; de Jong et al., 2010; Maratos, 2011). As these discrete emotion categories were presented equally often, frequency cannot explain the different effects, supporting the interpretation that it is the emotionality of the stimuli that gives rise to the differences. Furthermore, even though the present sample was relatively large, it could be argued that a larger spread in trait empathy could have helped in detecting more associations. In light of this, the lack of a correlation with the behavioral performance must, as with all null findings, be interpreted with care. Future studies could preselect participants to ensure greater variation in empathy scores, select extreme groups, or even test patient populations known to show impaired interpersonal reactivity, for example, individuals with autism spectrum disorder (Clark et al., 2008; Winkielman et al., 2009). Finally, the present study does not disentangle whether heightened empathy-related emotional responding is primarily related to the social content, the valence, or arousal aspect of the images. There has been some initial evidence showing that high empathic individuals, compared to low empathic individuals, are indeed more sensitive to cues of social versus monetary reward (Gossen et al., 2013) and that empathy-related responding is influenced by the phyologenetic similiarity of the stimuli to humans (Westbury and Neumann, 2008). A more systematic variation and experimentally testing of these variables (i.e., valence, arousal/salience aspect, and semantic content of stimuli) within one study design might be a worthwhile approach for future studies.

To conclude, the present results very convincingly affirm our knowledge about the influence of emotion on the attentional blink with pictorial stimuli. Emotional stimuli are salient enough to reduce the attentional blink effect behaviorally and also elicit enlarged P3 amplitudes, indicative of enhanced stimulus consolidation. This effect is related to individual differences in self-reported empathy, suggesting that individuals scoring high in interpersonal social traits show enhanced processing of emotional stimuli. The correlation with empathy scores suggests that individuals with high empathy may also be more sensitive to emotions expressed by others in everyday situations. As the attentional blink paradigm tests for stimulus processing in stimulus conditions that are suboptimal for conscious target detection with very brief presentation time and embedding in streams of other stimuli, it may be that in difficult and potentially stressful situations high empathy enables better emotion detection and adequate responding.

## Conflict of Interest Statement

The authors declare that the research was conducted in the absence of any commercial or financial relationships that could be construed as a potential conflict of interest.
